# Altered expression of circadian clock gene, mPer1, in mouse brain and kidney under morphine dependence and withdrawal

**DOI:** 10.1186/1740-3391-4-9

**Published:** 2006-08-22

**Authors:** Xiaojia Wang, Yueqi Wang, Haoyang Xin, Yanyou Liu, Yuhui Wang, Hang Zheng, Zhou Jiang, Chaomin Wan, Zhengrong Wang, Jian M Ding

**Affiliations:** 1 West China Medical Center, Sichuan University, Chengdu, Sichuan 610041, China; 2 National Laboratory of Biotherapy and Chronobiology, Public Health Department of China, China; 3 School of Physics, Sichuan University, Chengdu, Sichuan, China; 4 Department of Physiology, Brody School of Medicine, East Carolina University, Greenville, NC, USA

## Abstract

Every physiological function in the human body exhibits some form of circadian rhythmicity. Under pathological conditions, however, circadian rhythmicity may be dusrupted. Patients infected with HIV or addicted to drugs of abuse often suffer from sleep disorders and altered circadian rhythms. Early studies in *Drosophila *suggested that drug seeking behavior might be related to the expression of certain circadian clock genes. Our previous research showed that conditioned place preference with morphine treatment was altered in mice lacking the *Period-1 *(*mPer1*) circadian clock gene. Thus, we sought to investigate whether morphine treatment could alter the expression of *mPer1*, especially in brain regions outside the SCN and in peripheral tissues. Our results using Western blot analysis showed that the mPER1 immunoreactivity exhibited a strong circadian rhythm in the brains of the control (Con), morphine-dependent (MD), and morphine-withdrawal (MW) mice. However, the phase of the circadian rhythm of mPER1 expression in the brains of MD mice significantly differed from that of the Con mice (p < 0.05). In contrast to mPER1 expression in the brain, the circadian rhythm of mPER1 immunoreactivity in the kidneys was abolished after morphine administration, whereas the Con mice maintained robust circadian rhythmicity of mPER1 in the kidney. Therefore, the effect of morphine on the circadian clock gene *mPer1 *may vary among different organs, resulting in desynchronization of circadian function between the SCN and peripheral organs.

## Introduction

Circadian rhythms are about-daily variations of physiological functions that are found in every living organism on earth ranging from bacteria to mammals. These daily rhythms are generated through the integration of the oscillatory expression of multiple circadian clock genes [1-3]. In mammals, circadian rhythms are regulated by the suprachiasmatic nucleus (SCN) of the hypothalamus. Neurons in the SCN generate self-sustained daily oscillations of gene expression and electrical activity with a period close to 24 hours [4]. The SCN keeps the circadian rhythms of different peripheral organs synchronized to each other as well as to the environmental light-dark cycle [5]. Although every mammalian cell is believed to express circadian clock genes, cells outside the SCN cannot maintain self-sustained circadian oscillation in the absence of the SCN [6].

Almost every physiological function in the human body exhibits some form of circadian rhythmicity. Under pathological conditions, however, the normal circadian rhythm may be disrupted. AIDS patients or frequent users of recreational drugs often suffer from sleep disorders and altered circadian rhythms. Drug addicts often doze off during the day and wander around the street at night. This altered circadian behavior makes rehabilitation more difficult as these drug-depended patients cannot keep a steady daily schedule. It was reported that opioids could modify light entrainment of the circadian pacemaker via direct effects on SCN electrical activity and regulation of the *period *(*Per*) genes [7]. An early study found that delta opioid agonists could modulate light-induced phase advances in hamsters [8]. In addition, it has been reported that morphine could shift the circadian rhythm of locomotor activity in mice [9]. It is well known that morphine can induce adaptive changes in the central nervous system leading to the drug dependence [10]. Although the exact mechanism underlying morphine dependence is not fully understood, it has been reported that morphine dependence and morphine withdrawal syndrome are associated with the alteration of circadian rhythms. Previous studies in *Drosophila *indicated that behavioral sensitization to cocaine might be related to the expression of the clock genes *Period*, *Clock*, *Cycle*, and *Doubletime *[11]. Recently, we reported that conditioned place preference and locomotor sensitization for morphine were altered in mice lacking the *Period-1 *(*mPer1*) gene [12,13].

The mammalian *Period1 *(m*Per1*) gene is a major participant in the molecular feedback loop that generates circadian rhythms and plays a role in the resetting of the SCN by light signals [14]. In sheep, *Per1 *expression follows circadian as well as seasonal rhythms, with higher values in the summer when the day length is longer [15]. In the mouse SCN, the circadian pacemaker involves a transcriptional feedback loop in which CLOCK and BMAL1 function as positive regulators, whereas the three *Period *(*mPer*) genes, m*Per1*, m*Per2*, and m*Per3*, are involved in negative feedback. Moreover, *mPer1 *expression can be induced in the SCN by a brief light pulse during the dark phase [16]. The expression of *mPer *genes is not restricted to the SCN. The *mPer *genes are expressed in various other brain regions and peripheral tissues.

Since drug abuse is known to alter the circadian rhythm of behavior, we sought to investigate whether morphine treatment could alter the expression of circadian clock genes, especially in brain regions outside the SCN and in peripheral tissues.

## Materials and methods

### Animals

Male BALB/C mice, 4–6 weeks old, were used in the experiments. Animals were housed under standard conditions of ambient temperature (22 ± 2°C), humidity (55 ± 10%), and light (12L:12D, lights on at 8:00) and were fed food and water ad libitum. All efforts were made to minimize the number of animals used and their suffering. All experiments were performed in accordance with international guidelines on the ethical use of animals.

### Conditioned place preference (CPP)

The CPP test was carried out in a two-chamber apparatus (15 cm wide × 30 cm long × 15 cm high) with a sliding partition that divided the main unit into two equal-sized chambers. The two chambers differed in floor: one was white with a textured floor, and the other was black with a smooth floor. When the sliding partition was raised, mice could move freely from one chamber to the other. When CPP measured, the partition was raised to 7 cm above the floor. Mice were assayed for the time spent in the two chambers of the apparatus in 15 minutes. The time that mice spent in the drug-paired chamber was used as the CPP score. Each mouse had three daily adaptation sessions followed by CPP training, when it was given a morphine injection paired with restraint in the white-floor chamber for 30 min or a saline injection paired with restraint in the black-floor chamber for 30 min.

### Experimental protocol

Mice were randomly divided into three groups of 42 animals: Control (Con), Morphine-dependent (MD), and Morphine-withdrawal (MW). During the three adaptation sessions, the natural preference of the mice (for the white-floor chamber) was recorded. From the 4^th ^day on, all mice were engaged in the basic CPP training for eight days. Mice were given morphine (MD and MW, 10 mg/kg) or saline (Con) subcutaneously at 10:00 and then confined to the white side of the apparatus for 30 min. On the following day, they were given saline at 10:00 and then confined to the black section for 30 min. This 2-day procedure was repeated four times. Measurement of CPP was conducted at 16:00 each day. On the 12^th ^day, the mice in the Con group and the MD group were sacrificed at 0:00, 4:00, 8:00, 12:00, 16:00, and 20:00 (7 animals per time point per group). The brains and kidneys of the sacrificed mice were prepared for later analysis by western blot and immunohistochemistry. Mice in the MW group underwent morphine withdrawal for 5 days. On the 6^th ^day of withdrawal, the CPP was measured, and 7 mice were sacrificed at each of 6 time points (0:00, 4:00, 8:00, 12:00, 16:00, and 20:00). The brains and kidneys of these mice were prepared for later analysis by western blot and immunohistochemistry, respectively.

### Protein isolation and Western blotting

Brains and kidneys from 5 of the 7 animals in each group were used for Western blotting. Whole brain and kidney homogenates were obtained as follows. Tissue samples were homogenized at 4°C in a solution containing 0.4 M NaCl, 20 mM HEPES, 1 mM EDTA, 5 mM NaF, 1 mM dithiothreitol, 0.3% Triton X-100, 5% glycerol, 0.25 mM phenylmethylsulfonyl fluoride, 10 mg/ml aprotinin, 5 mg/ml leupeptin, and 1 mg/ml pepstatin A. Homogenates were cleared by centrifugation (twice, 12 min each, 12,000 × *g*). Proteins were separated by electrophoresis through 8% polyacrylamide separating gels with 5% polyacrylamide stacking gels and then transferred to nitrocellulose membranes. Membranes were blocked with 5% bovine serum albumin in Tris-buffered saline containing 0.05% Tween 20 and then incubated with affinity-purified antisera to mPER1 (Santa Cruz Biotechnology, Inc, USA). Immunoreactive bands were visualized using antigoat immunoglobulin G secondary antisera and enhanced chemiluminescence detection. Signals were then scanned by a Storm 840 instrument and analyzed by Image-Quant 5.0 software.

### Immunohistochemistry

Brains and kidneys from 2 of the 7 animals in each group were used for immunohistochemistry. The brains and kidneys prepared from sacrificed mice were fixed in 10% paraformaldehyde. Subsequently, they were dehydrated and blocked in paraffin. Serial sections of 4 nm were cut and processed for HE staining and immunohistochemistry. Sections were cleared of paraffin, and endogenous peroxidases were blocked by incubation with 3% H_2_O_2 _and washed.

Sections of the brains were then incubated with rabbit serum for 15 min at ambient temperature. Subsequently, the sections were incubated overnight with a goat polyclonal anti-mPER1 antibody (Santa Cruz Biotechnology, Inc, USA, 1:100) at 4°C, followed by the addition of biotinylated rabbit anti-goat IgG secondary antibody (Jinshan, BJ, China).

Sections of the kidneys were incubated overnight with a rabbit polyclonal anti-mPER1 antibody (Santa Cruz Biotechnology, Inc, USA, 1:25) at 4°C. Then, the sections were incubated with horseradish peroxidase (HRP)-conjugated secondary antibody directed against the relevant species (Jinshan, BJ, China).

Immunohistochemistry staining was processed in accordance with the manufacturer's instructions and visualized by the use of diaminobenzidine (DAB) staining. Immunoreactivity was analyzed through image pro plus software (Media CY Company). For every section, the integral optical density (IOD) of every visual field was calculated.

### Statistics

Data were analyzed by Student's *t*-tests for group differences, by one-way ANOVA for time differences and group differences separately, and by two-way ANOVA for time and group differences. The time series data of mPER1 protein expression, which were obtained by immunohistochemistry analyzed through image pro plus software, were analyzed for circadian rhythmicity by the cosinor method [17]. The parameters of the cosinor, i.e. Amplitude (half the difference between the minimum and maximum of the fitted cosine function), MESOR (middle value of the fitted cosine curve representing the rhythm adjusted mean) and Acrophase (time of peak value of the fitted cosine function), were tested between the two different groups separately by the cosinor parameters test designed by Bingham et al. [18].

## Results

### CPP

**Figure 1:**
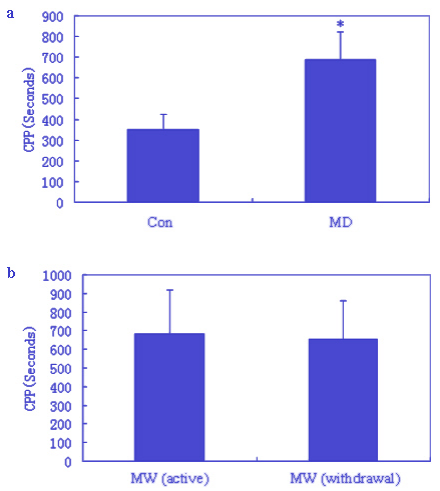
**The conditioned place preference (CPP) results**. Data of CPP in mice are given as mean (± S.E.M.) under the different conditions. **a**: CPP in the Con and MD groups (Con group after the 8^th ^day of saline injection, MD group after the 8^th ^day of morphine injection, * p < 0.05 tested by Student's *t*-test) **b**: CPP in the MW group (MW group after the 8^th ^day morphine injection and after 5^th ^day of morphine withdrawal).

During the three adaptation days, mice of neither group displayed a preference for the white or black chambers. After the 8^th ^day of morphine injection, MD and MW mice exhibited a preference for the morphine compartment, whereas the Con mice exhibited no preference for either compartments The mean CPPs of Con and MD mice were significantly different (Figure 1a). The CPP of MW mice on the 6^th ^day of withdrawal did not differ from that on the 8^th ^day of morphine administration (Figure 1b).

### Western blot for mPER1 protein

**Figure 2:**
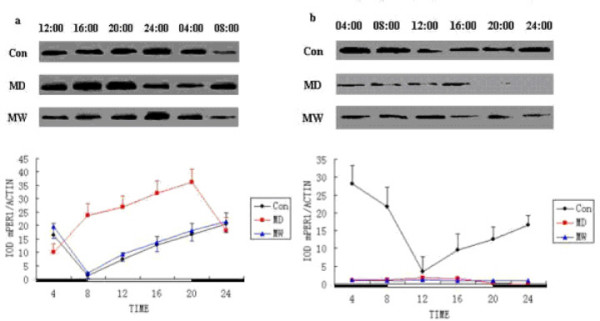
**The mPER1 protein expression levels of mice at the different time points**. Western blot analysis of the brains with anti-mPER1 polyclonal antibody reveals one distinct band at molecular weight of 110 kDa (Con, MD and MW, respectively). **a**: *Top*: mPER1 protein expressed in brains of mice. *Bottom*: data for mPER1 protein level were obtained by computerized analysis of the Western blots. Each value is the mean ± SEM. **b**: *Top*: mPER1 protein expressed in the kidneys of mice. *Bottom*: data for mPER1 protein level were obtained by computerized analysis of the Western blots. Each value is the mean ± SEM.

Western blot analysis of Con, MD and MW mouse brains and kidneys with anti-mPER1 goat polyclonal antibody revealed one distinct band at 110 kDa, which corresponds to mPER1 (Figure 2). Western blot test showed that the mPER1 protein, which reflects *mPer1 *gene expression, exhibited robust circadian rhythmicity in whole brain. The mPER1 protein expression level in MD mice was increased between 8:00 and 20:00. In Con and MW mice, high level of mPER1 protein expression in brains was observed at 0:00. Therefore, the phase of the circadian rhythm of *mPer1 *expression was advanced in mice of the MD group compared with the Con and MW groups (Figure 2a). Western blot test also showed that the mPER1 protein exhibited robust circadian variation in the kidneys of Con mice (Figure 2b). In contrast, there were weak expressions of mPER1 in the kidneys of the MD and MW mice.

### Immunohistochemical analysis of mPER1

**Figure 3:**
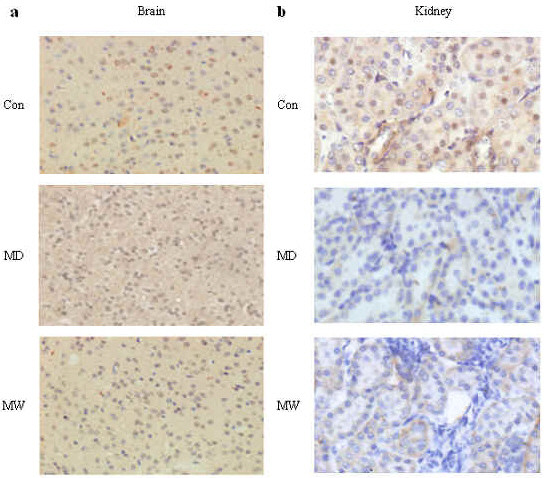
**Immunohistochemical stain for determining mPER1 protein expression in brains and kidneys**. **a**: Positive staining in the nucleus and cytoplasm are found in brains of Con, MD and MW mice. **b**: Representative cases show positive staining for mPER1 in kidneys of Con, MD and MW mice. Original magnification: 200× for all cases.

**Figure 4:**
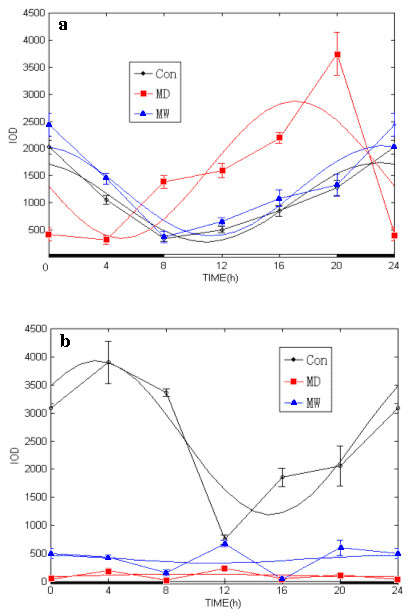
**Circadian variation of mPER1 protein expressed in the brains and kidneys of Con, MD and MW mice**. The integral optical density (IOD) of mPER1 immunoreactivity, an index of mPER1 protein expression level, was analyzed by image pro plus software. Time point means and SE of protein expression are shown along the 24-hour time scale. The best fitting cosine curves are shown in these panels. **a**: The mPER1 protein expression in brains was increased and acrophase of circadian rhythm was advanced in the MD mice as compared with Con and MW mice, statistically tested by the cosinor parameter test designed by Bingham et al. [18]. **b**: The mPER1 protein expression in the kidneys was severely inhibited and the circadian rhythm of mPER1 protein expression in the MD and MW mice was obliterated by morphine administration. Con mice exhibited robust rhythmicity in mPER1 expression.

Under high power (200×) and viewed with inverted microscope (Nikon TE 2000-U), mPER1 protein expression in the brains and kidneys were clearly observed. High expression of mPER1 is seen as brown-yellow, whereas low expression is seen as blue in the sections (Figure 3). Rhythmic expression of mPER1 was analyzed according to the mPER1 expression data determined by image pro plus software and shown in Figure 4 and Table 1.

**Table 1:** Cosinor analysis of mPER1 expression.

Group	P	MESOR ± SE (IOD)	Amplitude ± SE (IOD)	Acrophase (95 %CL) Hour
Expression of m PER1 in the brains				
Con	< 0.001	1010.8 ± 47.2	728.6 ± 66.8	-343.5° (-333, -354) 22:54
MD	< 0.001	1609.5 ± 149.9*	1263.1 ± 212.0*	-256.1° (-237, -275)* 17:04
MW	< 0.001	1221.6 ± 66.6	832.6 ± 94.3	-348.6° (-335, 0) 23:24
Expression of mPER1 in the kidneys				
Con	< 0.001	2559.2 ± 110.3	1368.4 ± 156.1	-47.7° (-34, -60) 03:11
MD	0.538	113.1 ± 15.9#	25.4 ± 22.5#	-148.5° (0, 0)# 09:54
MW	0.602	396.8 ± 45.9#	66.0 ± 64.9#	-350.5° (0, 0)# 23:22

* p < 0.05 compared with control or MW groups, separately, tested by the parameters of cosinor designed by Bingham et al. [18]. ^# ^p < 0.05 compared with control group, tested by the parameters of cosinor. The mPER1 protein expression level is represented by the IOD, which was the value of immunoreactivity of mPER1 in tissues reacting with mPER1 antibody determined by image pro plus software.

Con: Control; MD: Morphine-dependent; MW: Morphine-withdrawal. P in the table is the p-value of circadian rhythm coming from cosine function fitting. The hour in the table is the time of clock hour for the acrophase of the fitted cosine function.

Using immunohistochemistry and image analysis for expression of mPER1 protein, we found that the expression of mPER1 protein in the piriform cortex, nucleus accumbens and gyrus dentatus of the hippocampus fluctuated throughout the 12L:12D cycle (Figure 4a, Table 1). Circadian rhythmicity of mPER1 expression persisted after morphine administration, but the circadian pattern of mPER1 expression in the brains was changed: the MESOR was elevated and the acrophase (peak time) was shifted ahead in MD mice as compated to Con and MW mice. The acrophase of mPER1 expression did not differ significantly between the Con group (22:54) and the MW group (23:24). The acrophase was much earlier, however, in the MD group (17:04), as confirmed by the cosinor test.

Circadian variation of mPER1 protein expression was also observed in the kidneys of Con group mice, but not of MD and MW mice (Figure 4b, Table 1). In Con mice, mPER1 protein expression showed a peak at 3:11, whereas the peak value of mPER1 protein expression was not obviously noticed after morphine administration. The circadian expression of mPER1 protein was severely damped in the MD and MW mice compared with Con. The expression of mPER1 in the kidneys in Con, but not in MD and MW, showed statistically significant circadian rhythmicity (Figure 4b, Table 1).

## Discussion

Circadian rhythmicity is a highly conserved biological function that is found in every living organism from bacteria to humans. In mammals, circadian rhythms are regulated by the central circadian pacemaker in the SCN. In order for the organism to adapt to the environment, the circadian rhythms must be synchronized to the environmental light-dark cycle. This synchronization process is known as light entrainment, which occurs through daily light-induced phase advances and delays of the endogenous clock [19]. The SCN receives direct retinal input through a specialized subpopulation of light-sensitive but image forming-independent retinal ganglion cells that contain the photopigment melanopsin [20]. These ganglion cells project to the SCN and release glutamate and the neuropeptide pituitary adenylyl cyclase activating peptide (PACAP) as the principal neurotransmitters for light entrainment [21].

In order to optimize the bodily function of different organ systems, the SCN keeps the circadian rhythms of different peripheral organs synchronized to each other. For example, the catecholamine and the glucocorticoid hormone levels are high during the day when cardiovascular output is in high demand. During sleep, circulating lymphocytes reach the peak level to conduct immune surveillance. However, under pathological conditions, the circadian rhythms among different organ systems may not be well synchronized to each other, or to the environmental light-dark cycle. The results of the present study indicate that morphine treatment can abolish the circadian oscillation of mPER1 protein in the kidney and alter the phase of the oscillation in the brain. These results strongly suggest that morphine addiction and withdraw may lead to desynchronization of circadian rhythm between different organs.

Besides playing a role in regulating circadian rhythms, the role of *mPer1*, if any, in the brain and peripheral tissue is largely unknown. Our Western blot analysis using the whole brains revealed that the phase of the circadian rhythm of mPer1 was advanced in the morphine withdrawal mice compared to the control mice. In future studies, we will isolate brain structures that are known to be involved in drug addiction, including the limbic system, the dopaminergic neurons in the nucleus accumbens, and the arcuate nucleus, etc.

The exact role of the circadian clock genes in peripheral tissues remains unknown. Our results revealed that morphine treatment can abolish the circadian oscillation of mPER1 protein in the kidney and alter the phase of the oscillation in the brain. A previous study reported that morphine and its metabolites were secreted by the kidney after detoxification in the liver [22]. It was also reported that opiate addiction could result in renal diseases, including interstitial nephritis, glomerular epithelial cell apoptosis, nephrotic syndrome or acute renal failure [23-26]. Chen *et al*. [27] reported that urinary water excretion, sodium excretion and potassium excretion exhibit circadian rhythms in the rats, with peak activity occurring at night. Our results showed that the expression of mPER1 in the kidneys was higher at night in the control mice, coinciding with the peak activity of potassium excretion [27].

The SCN may regulate the circadian rhythms of peripheral organs through diverse pathways. A previous study reported that circadian rhythms of clock genes including *mPer1 *were maintained in the kidneys of SCN-lesioned mice [28]. In feeding studies, it was found that feeding schedules could entrain the circadian rhythm of clock gene expression in the liver independent of the SCN [29,30]. These findings suggest that the circadian rhythms of peripheral organs may be synchronized by nutrients or metabolic products, in addition to the SCN.

In summary, the effects of morphine on the circadian clock gene, *mPer1*, seem to be organ specific. In the brain, morphine increases the level of mPER1 expression and advances the phase of the circadian rhythm. In the kidney, morphine decreases the level of mPER1 expression and abolishes circadian rhythmicity.

## Competing interests

The author(s) declare that they have no competing interests.

## Authors' contributions

XW participated in all of the work and drafted the manuscript. YW participated in experiment designing. HX participated in data analysis. YL and CW participated in the CPP experiment. YW, HZ and ZJ participated in immunohistochemistry and western blot. JMD helped with the English writing of the paper. ZW directed the study and wrote the final version of the manuscript. All authors read and approved the final version of the article.

## References

[B1] Harmer SL, Panda S, Kay SA (2001). Molecular bases of circadian rhythms. Annu Rev Cell Dev Biol.

[B2] Dvornyk V, Vinogradova O, Nevo E (2003). Origin and evolution of circadian clock genes in prokaryotes. Proc Natl Acad Sci USA.

[B3] Merrow M, Spoelstra K, Roenneberg T (2005). The circadian cycle: daily rhythms from behavior to genes. EMBO Rep.

[B4] Herzog ED, Schwartz WJ (2002). A neural clockwork for encoding circadian time. J Appl Physiol.

[B5] Dardente H, Cermakian N (2005). How many pieces to build a circadian clock?. Med Sci (Paris).

[B6] Fukuhara C, Tosini G (2003). Peripheral circadian oscillators and their rhythmic regulation. Front Biosci.

[B7] Vansteensel MJ, Magnone MC, van Oosterhout F, Baeriswyl S, Albrecht U, Albus H, Dahan A, Meijer JH (2005). The opioid fentanyl affects light input, electrical activity and Per gene expression in the hamster suprachiasmatic nuclei. Eur J Neurosci.

[B8] Tierno A, Fiore P, Gannon RL (2002). Delta opioid inhibition of light-induced phase advances in hamster circadian activity rhythms. Brain Res.

[B9] Marchant EG, Mistlberger RE (1995). Morphine phase-shifts circadian rhythms in mice: role of behavioral activation. Neuroreport.

[B10] Amini H, Ahmadiani A (2005). In vivo evidence for an increase in 5alpha-reductase activity in the rat central nervous system following morphine exposure. Int J Dev Neurosci.

[B11] Andretic R, Chaney S, Hirsh J (1999). Requirement of Circadian Genes for Cocaine Sensitization in *Drosophila*. Science.

[B12] Liu Y, Wang Y, Wan C, Zhou W, Peng T, Liu Y, Wang Z, Li G, Cornelisson G, Halberg F (2005). The role of mPer1 in morphine dependence in mice. Neuroscience.

[B13] Wang YQ, Zhou W, Liu YY, Liu YH, Peng T, Wang ZR (2004). The role of circadian gene Period1 in morphine reward in mice. Space Med Med Eng (Beijing).

[B14] Bae K, Jin X, Maywood ES, Hastings MH, Reppert SM, Weaver DR (2001). Differential functions of mPer1, mPer2, and mPer3 in the SCN circadian clock. Neuron.

[B15] Shiromani PJ, Xu M, Winston EM, Shiromani SN, Gerashchenko D, Weaver DR (2004). Sleep rhythmicity and homeostasis in mice with targeted disruption of mPeriod genes. Am J Physiol Regul Integr Comp Physiol.

[B16] von Gall C, Schneider-Huther I, Pfeffer M, Dehghani F, Korf HW, Stehle JH (2001). Clock gene protein mPER1 is rhythmically synthesized and under cAMP control in the mouse pineal organ. J Neuroendocrinol.

[B17] Halberg F (1980). Chronobiology: Methodological problem. Act Med Rom.

[B18] Bingham C, Arbogast B, Cornellissen G, Lee JK, Halberg F (1981). Inferential statistical methods for estimating and comparing cosinor parameter. Chronobiologia.

[B19] Roenneberg T, Daan S, Merrow M (2003). The art of entrainment. J Biol Rhythms.

[B20] Van Gelder RN (2005). Nonvisual ocular photoreception in the mammal. Methods Enzymol.

[B21] Hannibal J, Moller M, Ottersen OP, Fahrenkrug J (2000). PACAP and glutamate are co-stored in the retinohypothalamic tract. J Comp Neurol.

[B22] Dubs A, Wiedemeier P, Caduff B (1999). Morphine poisoning in chronic kidney failure. Morphine-6-glucuronide as a pharmacologically active morphine metabolite. Dtsch Med Wochenschr.

[B23] Atici S, Cinel I, Cinel L, Doruk N, Eskandari G, Oral U (2005). Liver and kidney toxicity in chronic use of opioids: an experimental long term treatment model. J Biosci.

[B24] Dettmeyer RB, Preuss J, Wollersen H, Madea B (2005). Heroin-associated nephropathy. Expert Opin Drug Saf.

[B25] Patel J, Manjappa N, Bhat R, Mehrotra P, Bhaskaran M, Singhal PC (2003). Role of oxidative stress and heme oxygenase activity in morphine-induced glomerular epithelial cell growth. Am J Physiol Renal Physiol.

[B26] Stratta P, Canavese C, Messina M, Colla L, Dogliani M, Vercellone A (1986). Postpartum acute renal failure in a drug addict. Drug Alcohol Depend.

[B27] Chen LG, Wang ZR, Wan CM, Xiao J, Guo L, Guo HL, Cornelissen G, Halberg F (2004). Circadian renal rhythm influenced by implanted encapsulated hANP-producing cells in Goldblatt hypertension rats. Gene Ther.

[B28] Guo H, Brewer JM, Champhekar A, Harris RB, Bittman EL (2005). Differential control of peripheral circadian rhythms by suprachiasmatic-dependent neural signals. Proc Natl Acad Sci USA.

[B29] Stokkam KA, Yamazaki S, Tei H, Sakaki Y, Menaker M (2001). Entrainment of the circadian clock in the liver by feeding. Science.

[B30] Oishi K, Kasamatsu M, Ishida N (2004). Gene- and tissue-specific alterations of circadian clock gene expression in streptozotocin-induced diabetic mice under restricted feeding. Biochem Biophys Res Commun.

